# Emergency medicine pharmacotherapy compromises accuracy of plasma creatinine determination by enzyme-based methods: real-world clinical evidence and implications for clinical practice

**DOI:** 10.3389/fmed.2023.1236948

**Published:** 2024-01-08

**Authors:** Regina Demlova, Sarka Kozakova, Michal Rihacek, Dana Buckova, Katerina Horska, Ondrej Wiewiorka, Lubos Boucek, Iveta Selingerova, Martina Podborska, Alena Korberova, Alena Mikuskova, Jiri Starha, Miroslava Benovska, Martin Radina, Michal Richter, Lenka Zdrazilova Dubska, Dalibor Valik

**Affiliations:** ^1^Department of Pharmacology, Faculty of Medicine, Masaryk University, Brno, Czechia; ^2^Department of Pharmacy, University Hospital Brno, Brno, Czechia; ^3^Diagnostic and Therapeutic Centre, Emergency Services Department, University Hospital Brno, Masaryk University, Brno, Czechia; ^4^Department of Laboratory Medicine, University Hospital Brno, Masaryk University, Brno, Czechia; ^5^Department of Laboratory Methods, Faculty of Medicine, Masaryk University, Brno, Czechia; ^6^Department of Pharmacy, Clinical Pharmacy Services Unit, University Hospital Brno, Brno, Czechia; ^7^Department of Pharmacology and Toxicology, Faculty of Pharmacy, Masaryk University, Brno, Czechia; ^8^Department of Laboratory Medicine, University Hospital Brno, Brno, Czechia; ^9^Department of Mathematics and Statistics, Faculty of Science, Masaryk University, Brno, Czechia; ^10^Department of Pediatric Hematology and Biochemistry, University Hospital Brno, Brno, Czechia; ^11^Department of Pediatrics, Pediatric Nephrology Unit, University Hospital Brno, Brno, Czechia; ^12^Faculty of Medicine, Masaryk University, Brno, Czechia; ^13^Research Unit for Rare Diseases, Department of Pediatrics and Inherited Metabolic Disorders, First Faculty of Medicine, Charles University, Prague, Czechia; ^14^General Teaching Hospital, Prague, Czechia; ^15^Spadia Laboratories, Central Reference Lab, Division of Clinical Biochemistry, Ostrava, Czechia; ^16^Department of Pediatric Oncology, University Hospital Brno, Brno, Czechia

**Keywords:** creatinine, renal functions, eGFR, urgent care medication, cystatin C

## Abstract

**Background:**

Assessment of kidney function in emergency settings is essential across all medical subspecialties. Daily assessment of patient creatinine results from emergency medical services showed that some deviated from expected values, implying drug-related interference.

**Methods:**

Real-time clinical evaluation of an enzyme method (Roche CREP2) in comparison with the Jaffé gen. 2 method (Roche CREJ2) was performed. During the period of December 2022 and January 2023, we analyzed 8,498 patient samples, where 5,524 were heavily medicated STAT patient specimens, 500 were pediatric specimens, and 2,474 were from a distant general population in a different region using the same methods.

**Results:**

In 109 out of 5,524 hospital specimens (1.97%, *p* < 0.001), the CREP2 value was apparently (25% or more) lower than CREJ2. Suspect interfering medication was found in a sample of 43 out of 46 reviewed patients where medication data were available. This phenomenon was not observed in the general population.

**Conclusion:**

In a polymedicated urgent care hospital population, a creatinine enzyme method produces unreliable results, apparently due to multiple drug-related interferences.

## Introduction

Determination of blood creatinine is essential to assess kidney functional status. The first method used for creatinine measurement in clinical specimens was introduced by Jaffé (1). During the past two decades, Jaffé methods have been widely replaced by enzyme-based colorimetric methods employing sarcosine oxidase/peroxidase reaction chemistry (2). It has been shown repeatedly that enzymatic methods are susceptible to negative interference caused by several drugs, such as etamsylate (3), metamizole (4, 5), paracetamol, N-acetyl-p-benzoquinone imine (NAPQUI) (5), acetylcysteine (6), derivatives of salicylic acid (5, 7), high-dose acidum ascorbicum (8), and dobesilate (9). Negative interference by catecholamines at clinically relevant concentrations was also shown to affect these methods (10), and recently, monoclonal immunoglobulins have also been reported to interfere (11, 12). In addition, Roche SmPC for enzymatic creatinine determination (CREP2) states that methyldopa and levodopa cause artificially low results. Etamsylate, acetaminophen (paracetamol), acetylcysteine, and metamizole are also mentioned as potentially interfering substances (13).

Reviewing patient result sign-outs in a large tertiary care hospital performing up to 600 STAT creatinine determinations a day, we observed that approximately 2% of creatinine results deviated from preceding or expected creatinine values in the context of patient history and diagnosis, mode of hospital admission, reference ranges, and/or delta checks. The falls in measured creatinine levels exhibited unpredictable behavior independent of diagnosis—from trauma emergency services through general surgery, urology, and obstetrics to neurology, general internal medicine, and subspecialties such as cardiology, nephrology, and oncology. Of the drugs frequently used in these emergency settings, metamizole, paracetamol, etamsylate, and acetylcysteine represent common medications known to interfere with enzymatic creatinine determination. Observing these inconsistencies, we realized an imminent occurrence of a diagnostic and/or medication error problem, the basis of which may be uncontrollable drug-induced effects leading to falsely low creatinine values by the enzyme method followed by spuriously high eGFR calculated values.

Here, we report on retrospective evaluation of the affected method (CREP2), comparing its performance to a Jaffé generation 2 method (CREJ2) in the context of real-life emergency medicine pharmacotherapy and immediate implementation of corrective measures restoring the accuracy of eGFR determinations. To the best of our knowledge, this is the first report addressing inaccuracies of enzyme-based creatinine methods relevant to emergency medical services pharmacotherapy and providing immediate, clinically feasible corrective and preventive actions applicable worldwide for hospital emergency laboratory services.

## Materials and methods

### Survey setting and patient population

The study was performed as an exploratory survey based on pharmacovigilance signals on the potential influence of medications on laboratory tests. The aim was to identify and minimize potential risks from patient medications. The survey was approved by the multicentric IRB of University Hospital Brno under the number 03-091122/EK. Creatinine measurements were performed as a single additional analysis of all accessible clinical specimens covered by legally required informed consent. All methods were applied as described by the manufacturer. All participating laboratories were accredited under the ISO15189 standard. Central and pediatric laboratories of the University Hospital Brno and a Central reference laboratory Spadia lab, a.s. were involved. Altogether, 8,498 paired, real-time patient creatinine measurements were performed. Of those, 5,524 were STAT determinations at the Department of Laboratory Medicine, University Hospital Brno (A), 500 measurements at the Children’s Hospital laboratories (B), and 2,474 at Spadia lab a.s. (C).

(A) Hospital adult urgent care population: STAT creatinine determinations were analyzed by both CREP2 and CREJ2 methods summing up to 5,524 measurements from 21 December 2022 to 19 January 2023. This population consisted of 3,449 patients, 45.21% male patients and 54.79% female patients, with an age range of 18–99 years and a median age of 62 years.

(B) Hospital pediatric population: Five hundred STAT creatinine determinations were analyzed using both methods from 15 January 2023 to 9 February 2023. The population at the Children’s Hospital is mostly constituted of five intensive care units (neonatal, general pediatric, resuscitation care, infectious diseases, and pediatric oncology/transplantation). This population consisted of 317 patients, 54.9% male patients and 45.1% female patients, with an age range of 0–24 years and a median age of 8 years.

(C) Common adult population: From 5 January 2023 to 31 January 2023, we performed 2,474 measurements in a different geographic region (North Moravia, Czechia) using identical equipment and methods (Roche c702). This dataset served as a “field verification set” performed at the same time in an approximately 200 km distant population assumingly not taking medications used in urgent care hospital settings. This patient population was a general regional outpatient population unrelated to a University Hospital Brno referral population. This population consisted of 2,474 patients, 42.3% male patients and 57.7% female patients, with an age range of 1–99 years and a median of 60 years.

### Laboratory methods

Creatinine and cystatin C measurements were performed using Roche platforms Cobas 8,000, Pure, or Integra. The CREP2 (enzyme-based) and CREJ2 (rate-blanked and compensated Jaffé gen. 2) methods were installed on the Cobas 8,000 c702 serving as the main measurement device in our hospital. All methods were conducted as described by the manufacturer. Creatinine methods were calibrated using C.f.a.s. Roche IDMS-traceable calibration. Pediatric STAT creatinine determinations were performed at the Children’s Hospital using Roche Cobas Integra 400+ instruments as recommended by the manufacturer.

### Statistical analysis

The Bland–Altman plot was used to define the limits of agreement between the values of creatinine concentration with a 95% confidence interval. Scatterplots with identity lines were used to visualize log-transformed values of paired measurements. The chi-square test for equality of proportions was used to assess the significance of differences among the populations analyzed. Statistical significance was defined as a value of *p* of <0.05. Statistical analyses were performed using R Statistical Software (v4.2.2; R Core Team 2021) (14–16). The datasets generated and analyzed in the current study are not publicly available as they are based on individual patient measurements. The ISO15189-accredited laboratories store the primary data as per the requirement of this document.

## Results

### Data analysis

**Figure 1:**
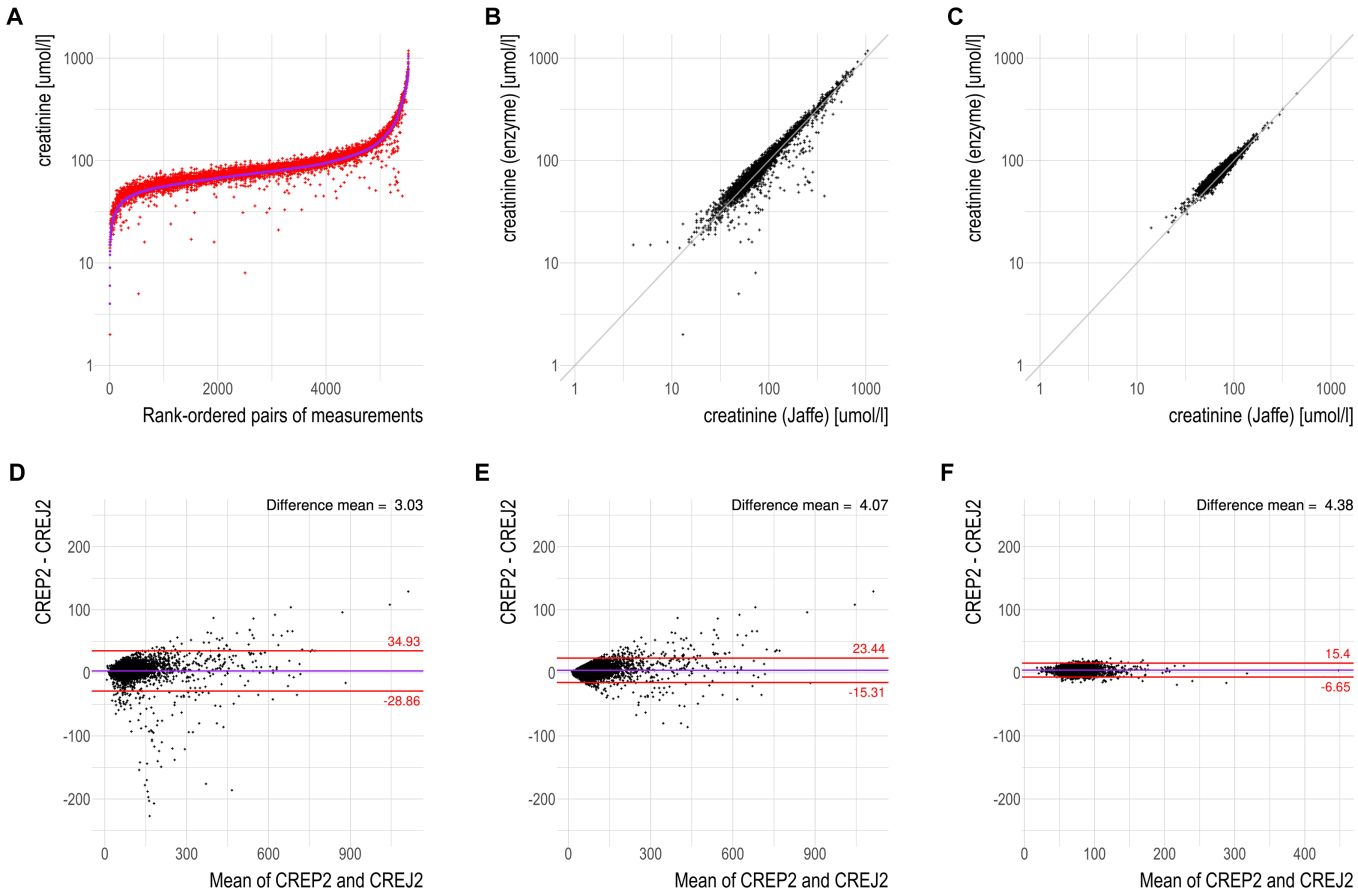
CREP2 exhibits a strong susceptibility to underestimate creatinine concentrations in the “hospital adult urgent care population” in contrast to the “common adult population.” **(A)** Logarithmic-scale scatterplot with paired data for the dataset “hospital adult urgent care population” with the samples being rank-ordered by creatinine concentrations determined by the CREJ2 method. The data show that CREP2 values were much lower than CREJ2 values in approximately 2% of the samples. **(B)** Logarithmic-scale scatterplot with identity line for the same dataset as in panel **(A)**. The identity line represents perfect agreement between the two methods; no negative interferences in the urgent care cohort with the CREJ2 method were observed. **(C)** Logarithmic-scale scatterplot with identity line for the dataset “common adult population” showing agreement between the two methods. **(D)** Bland–Altman plot for dataset “hospital adult urgent care population” from panel **(A)**; the horizontal solid line represents the mean difference between the two values, whereas the red (dotted) lines represent the limits of agreement (1.96 SD) of individual differences. **(E)** Bland–Altman plot for the same dataset as in panel **(D)** where outlying observations greater than 25% were removed. **(F)** Bland–Altman plot for dataset “common adult population” where no deviations between the two methods in the general community patient cohort were observed.

**Figure 2:**
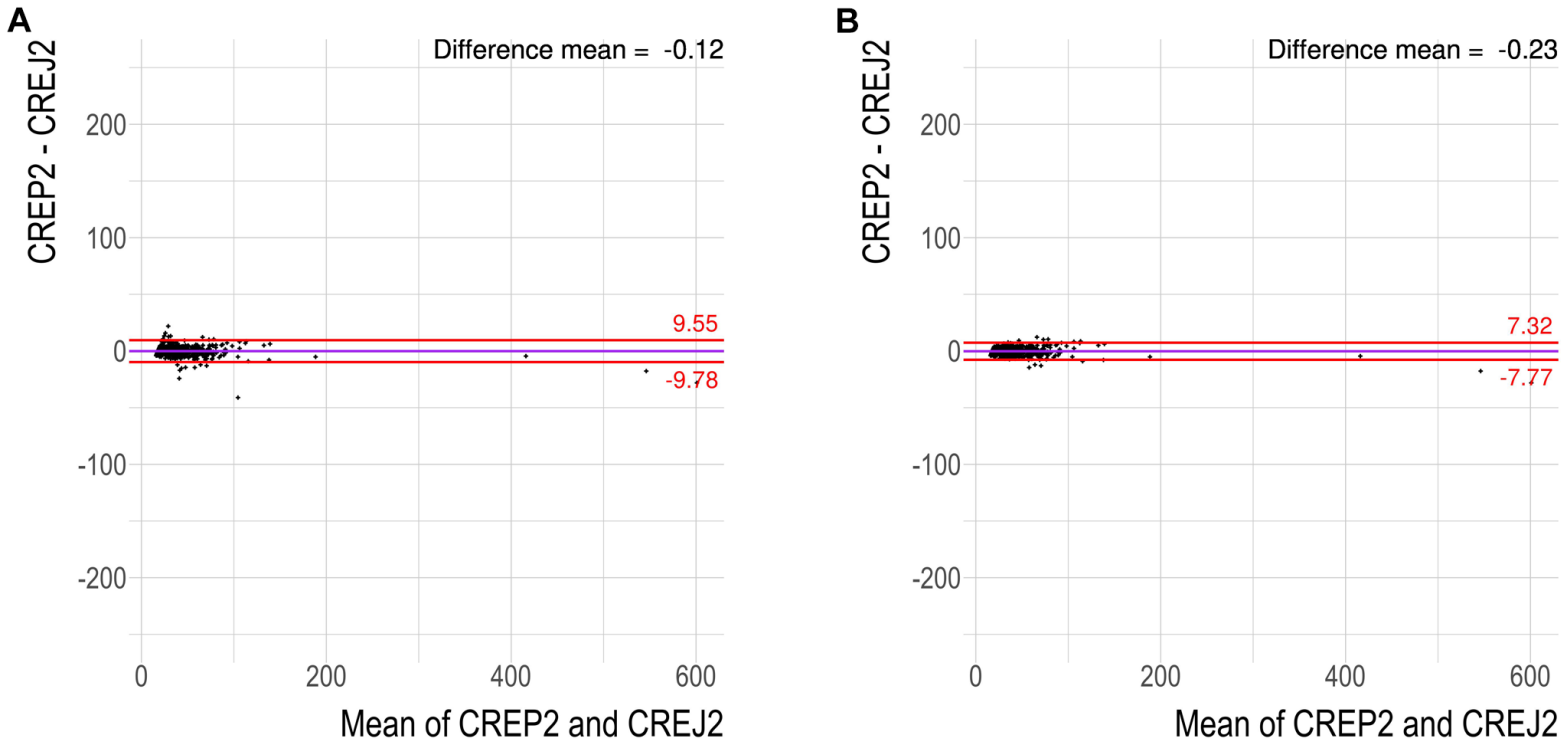
Pediatric hospital population does not exhibit susceptibility to underestimate creatinine concentrations by CREP2. **(A)** Bland–Altman plot for dataset “pediatric hospital population” showing 25 of 500 values greater than 1.96 SD. These cases were then individually screened by a pediatric nephrologist (JS) and a specialist in pediatric laboratory medicine (DV) for suspect medication. **(B)** Bland–Altman plot for the same dataset with any samples exhibiting the difference between the two methods greater than 25% removed.

**Figure 3:**
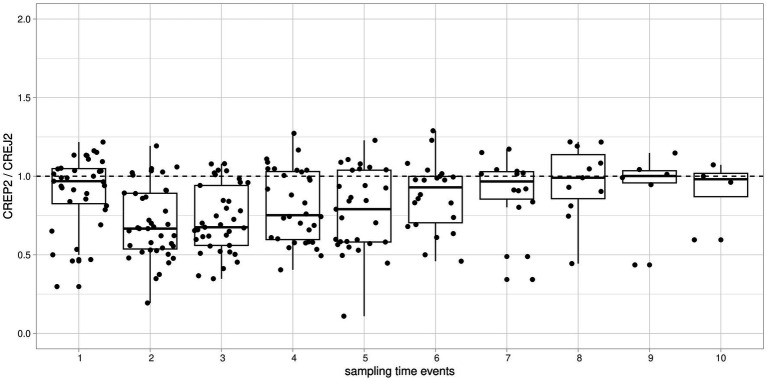
Time-dependent individual patient trends of consecutive creatinine measurements expressed as CREP2/CREJ2 ratio and aggregated and ranked to “sampling time events” from the first event through the tenth event. To visualize individual time-dependent trends of ratios of creatinine concentrations, we used relative timescale aggregating measurements as ordinal data. Boxplots of all patients/all measurements showed as “sampling time events 1 through 10” on the *x*-axis against the ratio of creatinine concentrations by CREP2/CREJ2 are shown. Major deviations from the median line observed among the 2nd through 7th measurement events implying a drug effect present but then returning to result concordance showed as a ratio approximately 1 implying a drug effect being “washed out.”

We used logarithmic transformation of the rank-ordered creatinine concentrations (x-axis) determined by the CREJ2 method (the lowest value first) and CREP2 methods to present all datapoints for population (A) as shown in Figure 1, Panel A. A substantial portion of CREP2 values deviates from a central tendency set by a blue sigmoid line of increasing CREJ2 concentrations. Panel 1b presents both creatinine methods as log-transformed data. The Panel 1d shows Bland–Altman plots of the full dataset as from Panel A1 visualizing a dispersion of creatinine concentrations. Panel 1e shows the identical dataset but with 25% of outlying values eliminated. Panels 1c and 1f describe the irrelevant general population (C) showing log-transformed values and a Bland–Altman plot showing very good agreement between the two methods. The results for the pediatric population are presented in Figure 2 as full data Bland–Altman plot (2a left panel) and after elimination of outliers greater than 25% (2b right panel). Figure 3 shows ratios of paired creatinine measurements as extracted from the table ranked from the first/s/third event (hospital admission > next morning sampling > next day sampling, etc.) through the tenth event (i.e., last measurements) as available. Interquartile ranges and medians are shown.

### Hospital patient record review

The clinical signal for this observational study was derived retrospectively from daily sign-out reviews. Values of CREP2 discrepant to CREJ2 with preceding measurements higher than 25% by CREP2 were first screened by two clinicians and two clinical pharmacists (MR and DB; KH and SK). We examined the use of etamsylate, metamizole (dipyrone), acetylcysteine, paracetamol, catecholamines, high-dose acidum ascorbicum, and dobesilate during the 72-h period before the time of collection of a sample with a suspiciously low CREP2 value. In urgent care patients, we reviewed drug administration timing in the context of creatinine concentrations that were extracted from daily LIMS results. The criteria were either delta checks and/or inconsistent patient result history. We identified the concerned medication in a sample of 43 of 46 reviewed patients where medication data were available for post-hoc access. All cases identified on the basis of 25% differences between creatinine measurements and selected for review are presented in Table 1. Figure 3 shows the evolution of discrepancies during the hospital stay between the two methods. Creatinine values usually were similar by both methods in the first sample collected after patient entry. With subsequent samples, the enzyme method tended to give low values, presumably because intensive drug treatment had started. With longer treatment, the difference between the methods became smaller, presumably because drug treatment had ended.

**Table 1:** Clinical cases showing blood creatinine concentrations against patient emergency pharmacotherapy timeframe.

Hospital Department, where the adverse event was detected	Patient/date	CREP2 (μmol/l)	CREJ2 (μmol/l)	Relevant medications (active substances) from patient discharge summaries (dosage unavailable) and/or daily medication cards (dosage available)
	1 (LR)	CREP2	CREJ2	
Department of Surgery	10 January 2023 12 January 2023 14 January 2023 15 January 2023 16 January 2023 17 January 2023 18 January 2023 19 January 2023 20 January 2023	186	N/A	Paracetamol 1 g orally every 8 h
		201	N/A	
		131	N/A	
		187	325	
		261	355	
		304	311	
		294	288	
		289	N/A	
		257	254	
	2 (NN)	CREP2	CREJ2	
Department of Surgery	11 January 2023 12 January 2023 13 January 2023 14 January 2023 15 January 2023 16 January 2023	81	N/A	Paracetamol 1 g/100 mL i.v. every 8 h (20 min), etamsylate 500 mg i.v. every 8 h
		92	N/A	
		34	N/A	
		61	87	
		90	86	
		106	98	
	3 (AE)	CREP2	CREJ2	
Department of Surgery	15 January 2023 16 January 2023 17 January 2023 24 January 2023	124	264	Paracetamol, metamizole, etamsylate 500 mg i.v. every 4 h
		127	220	
		89	177	
		135	130	
	4 (KL)	CREP2	CREJ2	
Department of Surgery	13 January 2023 14 January 2023 15 January 2023 16 January 2023 17 January 2023 18 January 2023 19 January 2023 20 January 2023 21 January 2023 22 January 2023	209	N/A	Etamsylate, noradrenalin continuous, paracetamol i.v., metamizole i.v.
		125	N/A	
		108	193	
		124	215	
		154	264	
		188	308	
		243	N/A	
		305	376	
		334	353	
		351	365	
	5 (LN)	CREP2	CREJ2	
Department of Infectious Diseases	15 January 2023 16 January 2023 19 January 2023 20 January 2023 21 January 2023	223	330	Metamizole, noradrenalin
		250	318	
		232	N/A	
		220	219	
		210	227	
	6 (AA)	CREP2	CREJ2	
Department of Anaestesiology and Intensive Care Medicine	10 January 2023 11 January 2023 12 January 2023	117	N/A	Etamsylate 500 mg i.v. every 6 h acetylcysteine inj. 300 mg i.v. every 12 h
		109	N/A	
		87	N/A	
	13 January 2023 14 January 2023 15 January 2023 16 January 2023 17 January 2023 18 January 2023	67	N/A	Noradrenalin 5 mg / 50 mL i.v. paracetamol 1 g i.v. 100 mL (20 min) every 6 h metamizole 1 g/20 mL (30 min)
		33	N/A	
		59	80	
		59	65	
		67	55	
		70	61	
	7 (AH)	CREP2	CREJ2	
Department of Pulmonary Diseases and Tuberculosis	11 January 2023 13 January 2023 15 January 2023	240	N/A	Etamsylate, paracetamol
		231	N/A	
		54	131	
	8 (NN)	CREP2	CREJ2	
Department of Internal Medicine and Cardiology	14 January 2023 15 January 2023 16 January 2023 18 January 2023 21 January 2023	184	N/A	Metamizole
		172	248	
		212	210	
		133	127	
		147	170	
	9 (YN)	CREP2	CREJ2	
Referred from another hospital	10 January 2023 11 January 2023 12 January 2023 15 January 2023	307	N/A	Etamsylate administered before patient transportation
		377	N/A	
		347	N/A	
		233	354	
	10 (KI)	CREP2	CREJ2	
Department of Burns and Plastic Surgery	10 January 2023 16 January 2023 17 January 2023 22 January 2023 27 January 2023	62	N/A	Etamsylate, noradrenalin, metamizole, paracetamol
		36	54	
		39	50	
		44	53	
		54	52	
	11 (AVA)	CREP2	CREJ2	
Department of Internal Medicine, Hematology and Oncology	14 January 2023 15 January 2023 16 January 2023 17 January 2023 19 January 2023 23 January 2023	51	N/A	Metamizole 500 mg orally every 8 h
		35	45	
		26	42	
		35	30	
		46	N/A	
		43	35	
	12 (KF)	CREP2	CREJ2	
Department of Neurosurgery	16 January 2023 17 January 2023 18 January 2023 19 January 2023 20 January 2023	50	100	Etamsylate
		73	85	
		74	77	
		77	N/A	
		86	79	
	13 (KRT)	CREP2	CREJ2	
Department of Anaestesiology and Intensive Care Medicine	14 January 2023 15 January 2022 16 January 2022 17 January 2023 18 January 2023 19 January 2023	97	N/A	Etamsylate 500 mg i.v. every 6 h, noradrenalin 5 mg/50 mL i.v., paracetamol 1 g/100 mL i.v. (30 min) as needed
		100	119	
		86	161	
		137	132	
		130	123	
		105	N/A	
	14 (KL)	CREP2	CREJ2	
Department of Geriatric Medicine	15 January 2023 16 January 2023 17 January 2023 18 January 2023 20 January 2023 23 January 2023	159	152	Etamsylate
		116	209	
		96	102	
		157	161	
		113	92	
		107	83	
	15 (LN)	CREP2	CREJ2	
Department of Internal Medicine and Cardiology	10 January 2023 11 January 2023 12 January 2023 13 January 2023 14 January 2023 15 January 2023 16 January 2023 17 January 2023	343	N/A	Metamizole, tramadol/paracetamol, acetylcysteine
		386	N/A	
		430	N/A	
		467	N/A	
		419	N/A	
		396	398	
		365	397	
		276	370	
	16 (AA)	CREP2	CREJ2	
	10 January 2023 17 January 2023 24 January 2023	47	N/A	Not accessible
		41	61	
		43	54	
	17 (KD)	CREP2	CREJ2	
Department of Neurology	14 January 2023 17 January 2023	102	N/A	Etamsylate
		51	82	
	18 (ATA)			
Department of Burns and Plastic Surgery	12 January 2023 12 January 2023 14 January 2023 17 January 2023 18 January 2023 18 January 2023 19 January 2023 23 January 2023 26 January 2023	62	N/A	Not accessible
		37	N/A	
		47	N/A	
		49	45	
		8	73	
		36	52	
		46	N/A	
		56	47	
		64	N/A	
	19 (OJ)	CREP2	CREJ2	
Department of Neurology	17 January 2023 18 January 2023 21 January 2023	100	108	Metamizole, paracetamol, etamsylate
		56	103	
		48	92	
	20 (AVA)	CREP2	CREJ2	
Department of Urology	18 January 2023 19 January 2023	79	68	Etamsylate, metamizole, paracetamol
		36	75	
	21 (SI)	CREP2	CREJ2	
Department of Urology	18 January 2023 19 January 2023 23 January 2023	106	97	Metamizole, etamsylate, paracetamol
		68	119	
		90	87	
	23 (IN)	CREP2	CREJ2	
Department of Burns and Plastic Surgery	13 January 2023 14 January 2023 15 January 2023	45	N/A	
		60	N/A	
		54	52	
	17 January 2023 19 January 2023 20 January 2023 22 January 2023 27 January 2023	45	49	Not accessible
		28	49	
		36	42	
		22	45	
		45	43	
	24 (YK)	CREP2	CREJ2	
Department of Burns and Plastic Surgery	13 January 2023 16 January 2023 18 January 2023 19 January 2023 20 January 2023 23 January 2023 24 January 2023 25 January 2023 27 January 2023	68	N/A	Not accessible
		59	68	
		52	77	
		65	N/A	
		39	69	
		84	85	
		69	68	
		56	46	
		49	47	
	25 (KL)	CREP2	CREJ2	
Department of Neurosurgery	18 January 2023 20 January 2023 21 January 2023 21 January 2023 22 January 2023 23 January 2023 24 January 2023 25 January 2023	95	84	Etamsylate, acetylcysteine, paracetamol
		77	118	
		94	98	
		91	92	
		102	98	
		81	83	
		107	93	
		91	84	
	26 (LLF)	CREP2	CREJ2	
Department of Internal Medicine and Gastroenterology	18 January 2023 19 January 2023 20 January 2023 21 January 2023 22 January 2023 23 January 2023 24 January 2023	406	358	Etamsylate
		276	N/A	
		284	431	
		273	448	
		267	448	
		345	415	
		391	379	
	27 (SN)	CREP2	CREJ2	
Department of Internal Medicine and Gastroenterology	18 January 2023 19 January 2023 20 January 2023 21 January 2023 22 January 2023 23 January 2023 24 January 2023 25 January 2023 26 January 2023 27 January 2023	74	81	Polymedication including terlipressin acetate
		69	N/A	
		48	78	
		59	67	
		38	85	
		79	81	
		102	87	
		102	103	
		95	N/A	
		89	83	
	28 (YEL)	CREP2	CREJ2	
Department of Urology	20 January 2023 (11:43) 20 January 2023 (17:05) 21 January 2023 22 January 2023 23 January 2023	442	640	
		362	583	
		304	587	
		263	492	
		228	324	
Department of Surgery	24 January 2023 (5:05) 24 January 2023 (17:08) 25 January 2023 26 January 2023 27 January 2023 28 January 2023 29 January 2023	294	333	Etamsylate 500 mg i.v. every 6 h, metamizole every 8 h
		317	379	
		330	355	
		366	N/A	
		429	429	
		462	474	
		494	496	
	29 (PŘI)	CREP2	CREJ2	
Department of Neurosurgery	20 January 2023 21 January 2023 22 January 2023 (6:07) 22 January 2023 (9:35) 23 January 2023 24 January 2023 25 January 2023	94	100	Metamizole 500 mg every 12 h, etamsylate
		80	88	
		42	85	
		82	113	
		67	112	
		49	98	
		100	96	
	30 (ATA)	CREP2	CREJ2	
	21 January 2023 22 January 2023 23 January 2023	78	93	Etamsylate 500 mg i.v. every 4 h
Department of Anaestesiology and Intensive Care Medicine		32	67	
		29	64	
	31 (AEL)	CREP2	CREJ2	
Department of Infectious Diseases Department of Urology	16 January 2023 24 January 2023 29 January 2023 30 January 2023 31 January 2023 01 February 2023	119	105	Etamsylate, paracetamol
		57	102	
		108	212	
		72	178	
		67	140	
		92	108	
	32 (KIR)	CREP2	CREJ2	
Department of Urology	23 January 2023 24 January 2023 25 January 2023 27 January 2023 29 January 2023	113	127	Polymedication including: ampicilin/sulbactam, bisoprolol, enoxaparine, methoclopramide, simeticone, omeprazole, itopride, alopurinol, pantoprazole, rosuvastatine, metformin, rivaroxaban, linesolide, bromazepam
		75	149	
		149	138	
		151	147	
		146	132	
	33 (YAV)	CREP2	CREJ2	
Department of Urology	23 January 2023 24 January 2023 25 January 2023 27 January 2023 29 January 2023	42	141	Metamizole, etamsylate, paracetamol
		75	167	
		105	168	
		117	154	
		111	118	
	34 (LRI)	CREP2	CREJ2	
Department of Urology	16 January 2023 23 January 2023 24 January 2023 25 January 2023 27 January 2023 29 January 2023	84	69	Metamizole, etamsylate, paracetamol
		90	87	
		46	77	
		66	63	
		50	86	
		77	77	
	35 (SAN)	CREP2	CREJ2	
	25 January 2023 (0:35) 25 January 2023 (6:17)	54	83	
		54	75	
Department of Anaestesiology and Intensive Care Medicine Department of Surgery	25 January 2023 (8:19) 26 January 2023 27 January 2023 30 January 2023	54	77	Etamsylate 500 mg i.v. every 4 h
		53	N/A	
		48	57	
		51	60	
	36 (AAV)	CREP2	CREJ2	
Department of Infectious Diseases	20 January 2023 23 January 2023 25 January 2023	133	142	Polymedication including: insuline-lispro, insuline-glargin, perindopril-arginine, linezolid, rosuvastatin, pantoprazol
		137	134	
		40	115	
	37 (HAN)	CREP2	CREJ2	
Department of Surgery	15 January 2023 21 January 2023 22 January 2023 23 January 2023 24 January 2023 25 January 2023 27 January 2023 28 January 2023 29 January 2023 30 January 2023	142	140	From 21 January 2023: etamsylate 500 mg i.v. every 6 h paracetamol 1 g i.v. every 8 h, noradrenalin continuous
		345	336	
		285	337	
		192	319	
		157	286	
		124	270	
		120	350	
		151	340	
		149	342	
		194	326	
	38 (KAV)	CREP2	CREJ2	
Department of Anaestesiology and Intensive Care Medicine	24 January 2023 25 January 2023 26 January 2023 27 January 2023 28 January 2023 29 January 2023	153	158	Etamsylate 500 mg i.v. every 4 h, noradrenalin continuous 10 mg / 50 ml i.v., paracetamol 1 g i.v. infusion
		145	207	
		239	N/A	
		179	244	
		145	155	
		127	125	
	39 (BCH)	CREP2	CREJ2	
Department of Pulmonary Diseases and Tuberculosis	23 January 2023 25 January 2023 30 January 2023	48	61	Etamsylate
		42	63	
		97	95	
	40 (ALA)	CREP2	CREJ2	
Department of Surgery	26 January 2023 27 January 2023 29 January 2023	147	N/A	27 January 2023: paracetamol 500 mg á every h i.v., etamsylate 500 mg every 4 h
		70	132	
		45	81	
	41 (KAV)	CREP2	CREJ2	
Department of Gastroenterology Department of Urology	19 January 2023 23 January 2023 26 January 2023 27 January 2023 28 January 2023 29 January 2023 30 January 2023	103	98	Paracetamol, etamsylate, erdosteine
		99	83	
		61	N/A	
		90	121	
		131	155	
		160	154	
		147	145	
	42 (TIR)	CREP2	CREJ2	
Department of Internal Medicine and Gastroenterology	25 January 2023 26 January 2023 27 January 2023 28 January 2023	455	N/A	
		541	N/A	
		410	593	
		500	673	
	29 January 2023 30 January 2023 31 January 2023 01 February 2023 02 February 2023	584	739	Not accessible
		649	782	
		625	685	
		494	531	
		569	572	
	43 (AIM)	CREP2	CREJ2	
Department of Neurosurgery	26.01.2023 27.01.2023 31.01.2023	73	N/A	Polymedication including: perindopril-erbumin 4 mg tbl. 1–0-0, omeprazole 20 mg tbl. 1–0-1, dexamethasone 4 mg tbl. 1–0-0, escinum tbl. 2–2-2, nadroparine 0.6 ml s.c. 1x daily
		45	87	
		69	64	
	44 (LAS)	CREP2	CREJ2	
Department of Internal Medicine, Geriatrics and Practical Medicine	21 January 2023 23 January 2023 26 January 2023 27 January 2023 (6:20) 27 January 2023 (8:57) 28 January 2023 29 January 2023 30 January 2023 31 January 2023	187	181	Paracetamol 500 mg 2–2-2 orally as needed, etamsylate 2amp. iv. every 4 hod. 5–9-13 h, noradrenaline
		146	145	
		125	N/A	
		91	157	
		82	155	
		106	167	
		142	177	
		177	196	
		218	220	
	45 (KES)	CREP2	CREJ2	
Department of Otorhinolaryngology	18 January 2023 19 January 2023 21 January 2023 25 January 2023 27 January 2023 01 February 2023	57	57	Not accesible
		60	N/A	
		61	62	
		56	44	
		31	53	
		59	68	
	46 (MAV)	CREP2	CREJ2	
Department of Internal Medicine, Geriatrics and Practical Medicine Department of Neurosurgery Department of Infectious Diseases	23 January 2023 25 January 2023 26 January 2023 27 January 2023 30 January 2023	129	112	Paracetamol 500 mg orally 1–1–1 as needed, metamizole 1 g intramuscularly 1–0–1
		134	N/A	
		102	N/A	
		74	127	
		151	140	
	47 (AOJ)	CREP2	CREJ2	
Department of Urology	26 January 2023 28 January 2023 01 February 2023	179	N/A	Not accessible
		150	285	
		106	232	
	48 (AUB)	CREP2	CREJ2	
Department of Trauma Surgery	28 January 2023 29 January 2023	102	103	Metamizole, etamsylate, paracetamol
		31	89	
	49 (AAR)	CREP2	CREJ2	
Department of Trauma Surgery	28 January 2023 29 January 2023	94	95	Paracetamol 1 g i.v. every 8 h
		18	93	
	50 (YAV)	CREP2	CREJ2	
Department of Internal Medicine and Gastroenterology	27 January 2023 28 January 2023 29 January 2023 (6:32) 29 January 2023 (16:54) 30 January 2023	305	296	Not accessible
		317	348	
		263	402	
		181	332	
		175	353	
	31 January 2023 01 February 2023 02 February 2023	206	303	
		217	273	
		263	296	
	51 (KIN)	CREP2	CREJ2	
Department of Trauma Surgery	29 January 2023 30 January 2023 31 January 2023	108	112	Etamsylate, paracetamol
		39	104	
		33	90	
	52 (LEF)	CREP2	CREJ2	
Department of Pulmonary Diseases and Tuberculosis	28 January 2023 29 January 2023 30 January 2023 31 January 2023 01 February 2023	747	721	Etamsylate
		646	610	
		151	202	
		81	73	
		66	59	
	53 (AAS)	CREP2	CREJ2	
	28 January 2023 29 January 2023 30 January 2023 01 February 2023 02 February 2023	100	117	Noradrenalin 10 mg / 50 mL continuous i.v.
Department of Anaestesiology and Intensive Care Medicine		97	109	
		98	146	
		143	183	
		151	154	
	54 (KIR)	CREP2	CREJ2	
Department of Internal Medicine and Gastroenterology	23 January 2023 25 January 2023 30 January 2023 31 January 2023 01 February 2023 02 February 2023	481	434	Paracetamol
		477	455	
		407	627	
		527	767	
		635	794	
		732	862	
	55 (YEL)	CREP2	CREJ2	
Department of Urology	30 January 2023 31 January 2023	47	102	Metamizole, etamsylate, paracetamol
		61	90	

Selection of cases was done on the basis of a 25% fall in preceding creatinine concentrations; data were taken as accessible from medical records.

### Result summary

In the hospital adult urgent care population (A), we found 109 samples (1.97%) where the CREP2 value was 25% or more below the CREJ2 value. There were no such samples in the common adult population (C). The proportions were significantly different (*p* < 0.001) between the population (A) and population (C). We conclude that in a polymedicated urgent care hospital population, the creatinine enzyme method produces unreliable results due to multiple drug-related interferences.

## Discussion

Inaccurate clinical inference on renal functions may severely affect diagnostic workup and threaten the safety and effectiveness of treatment. Among a number of putative filtration biomarkers, only cystatin C has been partially adopted in clinical practice, mostly in nephrology specialties, but not in emergency medical services (17). Despite common criticism, blood creatinine remains the only biomarker accessible worldwide. Therefore, the accuracy of its determinations is of paramount importance to reliably calculate eGFR that is predominantly recommended for clinical use (18). Although mentioned in SmPCs of the respective drugs (19–21) and a procedure manual of the affected method (22) as well, real-life urgent care practice often dictates an immediate clinical need for medication and, at the same time, renal function assessment. As we show here, enzyme methods applied on patient STAT specimens with multiple medications do not provide a reliable platform for creatinine determination. These findings led us to reintroduce the CREJ2 method to clinical practice as the main tool for creatinine determination. The Roche CREJ2 rate-blanked and compensated method has an IDMS-traceable calibration and is therefore suitable for eGFR calculations. Significant improvements in the original Jaffé method have been made, with this generation showing reduced interferences. We kept the CREP2 methods on our instrumentation available as needed, that is, double-checks, patients with muscular wasting, sarcopenia, pediatric population with creatinine less than 20 μmoL/L, and/or clinical trials with new drugs whose behavior toward creatinine determination is not known.

Clinical practice in our hospitals set new laboratory standards for the evaluation of renal functions. The screening tier in the adult population consists of (i) CREJ2 determination resulting in CKD-EPI eGFR calculation always available as STAT test. The second tier consists of (ii) CREJ2 + cystatin C determinations available STAT on a physician’s specific request resulting in a composite CKD-EPI (“DUO”) eGFR calculation—this procedure is intended as a quantitative measure of eGFR covering clinical situations where creatinine may be biologically unreliable, such as muscle wasting processes and extremity amputations. For pediatric populations, the screening tier is (iii) CREJ2 plus patient height resulting in Schwartz-bedside eGFR calculation, and (iv) the quantitative tier is urea+CREJ2 + cystatin C determination and eGFR calculation using the “full” Schwartz equation (23). This practice integrated our findings and general recommendations as well (24, 25).

## Conclusion

From a clinical point of view, treatment is always a priority, especially in the emergency setting. Diagnostic laboratories shall use methods suitable for the intended purpose such as in specific patient populations. However, it is the IVD manufacturer’s responsibility to provide such technologies declaring suitability for a given patient setting such as emergency medicine.

Regarding kidney function biomarkers, we think that combining both biological markers, creatinine and cystatin C, reduces the disadvantages associated with each of them separately. This may advance cystatin C usage to urgent care testing in large referral centers as they deal with polymedicated specimens from severely sick patients often with no medical history available. Our new practice greatly improved the reliability of renal function evaluation in our hospitals and the safety of diagnostic procedures and eGFR-based drug dosage as well. Although the nature of these drug-related interferences is not fully clear (26), our hypothesis is that drugs and/or drug-related substances with reducing chemical properties may interfere with the hydrogen peroxide/peroxidase step, thus severely affecting the reporter reaction.

### Limitations

Our report has several limitations. First, we did not mention which of the two methods produces “true” results. From a laboratory point of view, the *personalized and often temporary nature* of these abnormalities does not enable their detection by external means such as proficiency testing surveys, where both methods perform satisfactorily, nor by internal means such as delta checks in patients with *no medical record history*. Nevertheless, under our survey design, the CREJ2 method did not produce any false-negative results. Second, the lower limit of quantitation is higher for CREJ2 methods than enzyme methods, which is a limitation in pediatric medicine. Therefore, our pediatric algorithm for specimens <20 μmoL/L by CREJ2 implies that both methods are used; notably, in interference-free specimens, the CREP2 method showed excellent analytical performance, stability, and linearity down to 7 μmoL/L (data not shown). Third, it was not technically possible to timely screen all patient records for all possible medications, in part due to the legal inaccessibility of some records.

## Data availability statement

The raw data supporting the conclusions of this article will be made available by the authors, upon reasonable request.

## Ethics statement

The studies involving humans were approved by the University Hospital Brno, Multicentric Ethics Review Board, approval No. 03-091122/EK. The studies were conducted in accordance with the local legislation and institutional requirements. The human samples used in this study were acquired from a by-product of routine care or industry. Written informed consent for participation was not required from the participants or the participants’ legal guardians/next of kin in accordance with the national legislation and institutional requirements.

## Author contributions

RD conceived the topic, codesigned methods and pharmacovigilance issues, participated in the result interpretation and writing of the manuscript, was confirmed to have full access to all the data in the study, and accepted the responsibility to submit for publication. SK conceived the topic, codesigned methods, performed medication analysis, participated in result interpretation and writing of the manuscript, was confirmed to have full access to all the data in the study, and accepted the responsibility to submit for publication. MRih conceived the topic, performed interactions with clinical wards with documentation reviews and medication analyses, participated in the result interpretation and writing of the manuscript, was confirmed to have full access to all the data in the study, and accepted the responsibility to submit for publication. DB reviewed patient documentation, performed pediatric analyses with composite eGFR calculations such as Schwartz formulas, participated in the writing of the manuscript, was confirmed to have full access to all the data in the study, and accepted the responsibility to submit for publication. KH reviewed patient documentation, performed medication analyses, participated in the result interpretation and writing of the manuscript, was confirmed to have full access to all the data in the study, and accepted the responsibility to submit for publication. OW performed laboratory workups, participated in conceiving the topic and writing of the manuscript, was confirmed to have full access to all the data in the study, and accepted the responsibility to submit for publication. LB participated in the design of the survey and its conception, performed statistical analyses, participated in the result interpretation and writing of the manuscript, was confirmed to have full access to all the data in the study, and accepted the responsibility to submit for publication. IS surveyed statistical analyses, participated in the writing of the manuscript, was confirmed to have full access to all the data in the study, and accepted the responsibility to submit for publication. MP performed laboratory analyses, participated in the writing of the manuscript, was confirmed having full access to all the data in the study, and accepted the responsibility to submit for publication. AK performed clinical case analyses, participated in conceiving the manuscript and in result interpretation and writing of the manuscript, was confirmed to have full access to all the data in the study, and accepted the responsibility to submit for publication. AM performed pediatric laboratory analyses, participated in the writing of the manuscript, was confirmed to have full access to all the data in the study, and accepted the responsibility to submit for publication. MB performed laboratory analyses, participated in the writing of the manuscript, was confirmed to have full access to all the data in the study, and accepted the responsibility to submit for publication. JS coordinated laboratory practice for pediatric patients and participated in the writing of the manuscript, was confirmed to have full access to all the data in the study, and accepted the responsibility to submit for publication. MRa coordinated laboratory analyses in an outpatient population, participated in result interpretation and writing of the manuscript, was confirmed to have full access to all the data in the study, and accepted the responsibility to submit for publication. MRic performed laboratory analyses in an outpatient population, participated in the writing of the manuscript, was confirmed to have full access to all the data in the study, and accepted the responsibility to submit for publication. LZ conceived the topic, coordinated working activities, surveyed medication analyses with a focus on antimicrobial therapy, coordinated overall interpretations of findings and writing of the manuscript, was confirmed to have full access to all the data in the study, and accepted the responsibility to submit for publication. DV conceived the topic, coordinated all working activities, surveyed all analyses, coordinated final result interpretation, edited and coordinated the writing of the manuscript, was confirmed to have full access to all the data in the study, and accepted the responsibility to submit for publication. All authors contributed to the article and approved the submitted version.
